# Risk-group discrimination in node-negative breast cancer using invasion and proliferation markers: 6-year median follow-up

**DOI:** 10.1038/sj.bjc.6690373

**Published:** 1999-05-01

**Authors:** N Harbeck, P Dettmar, C Thomssen, U Berger, K Ulm, R Kates, H Höfler, F Jänicke, H Graeff, M Schmitt

**Affiliations:** Frauenklinik, Klinikum rechts der Isar, Institut für Allgemeine Pathologie und Pathologische Anatomie, andInstitut für Medizinische Statistik und Epidemiologie, Technische Universität München, Ismaningerstr. 22, D-81675 Munich, Germany; Universitätsfrauenklinik Eppendorf, Hamburg, Germany

**Keywords:** breast cancer, plasminogen activator inhibitor type 1 (PAI-1), prognosis, S-phase fraction, tumour biology, urokinase-type plasminogen activator (uPA)

## Abstract

Factors reflecting two major aspects of tumour biology, invasion (urokinase-type plasminogen activator (uPA), plasminogen activator inhibiter (PAI-1), cathepsin D) and proliferation (S-phase fraction (SPF), Ki-67, p53, HER-2/neu), were assessed in 125 node-negative breast cancer patients without adjuvant systemic therapy. Median follow-up time was 76 months. Antigen levels of uPA, PAI-1 and cathepsin D were immunoenzymatically determined in tumour tissue extracts. SPF and ploidy were determined flow-cytometrically, Ki‴-67, p53, and HER-2/neu immunohistochemically in adjacent paraffin sections. Their prognostic impact on disease-free (DFS) and overall survival (OS) was compared to that of traditional factors (tumour size, grading, hormone receptor status). Univariate analysis determined PAI-1 (*P* < 0.001), uPA (*P* = 0.008), cathepsin D (*P* = 0.004) and SPF (*P* = 0.023) as significant for DFS. All other factors failed to be of significant prognostic value. In a Cox model, only PAI-1 was significant for DFS (*P* < 0.001, relative risk (RR) 6.2). In CART analysis for DFS, the combination of PAI-1 and uPA gave the best risk group discrimination. For OS, PAI-1, cathepsin D, tumour size and ploidy were statistically significant in univariate, but PAI-1 was the only independently significant factor in Cox analysis (*P* < 0.001, RR 8.9). In particular, this analysis shows that PAI-1 is still a strong and independent prognostic factor in node-negative breast cancer after extended 6-year median follow-up. © 1999 Cancer Research Campaign


*British Journal of Cancer* (1999) **80**(3/4), 419–426
© 1999 Cancer Research Campaign
Article no. bjoc.1998.0373

### Risk-group discrimination in node-negative breast cancer using invasion and proliferation markers: 6-year median follow-up

**N Harbeck[1], P Dettmar[2], C Thomssen[4], U Berger[3], K Ulm[3], R Kates[3], H Höfler[2], F Jänicke[4], H Graeff[1] and M Schmitt[1]**

[1]Frauenklinik, Klinikum rechts der Isar, [2]Institut für Allgemeine Pathologie und Pathologische Anatomie and [3]Institut für Medizinische Statistik und Epidemiologie, Technische Universität München, Ismaningerstr. 22, D-81675 Munich, Germany; [4]Universitätsfrauenklinik Eppendorf, Hamburg, Germany

**Summary** Factors reflecting two major aspects of tumour biology, invasion (urokinase-type plasminogen activator (uPA), plasminogen activator inhibiter (PAI-1), cathepsin D) and proliferation (S-phase fraction (SPF), Ki-67, p53, HER-2/neu), were assessed in 125 node-negative breast cancer patients without adjuvant systemic therapy. Median follow-up time was 76 months. Antigen levels of uPA, PAI-1 and cathepsin D were immunoenzymatically determined in tumour tissue extracts. SPF and ploidy were determined flow-cytometrically, Ki‴-67, p53, and HER-2/neu immunohistochemically in adjacent paraffin sections. Their prognostic impact on disease-free (DFS) and overall survival (OS) was compared to that of traditional factors (tumour size, grading, hormone receptor status). Univariate analysis determined PAI-1 ($P < 0.001$), uPA ($P = 0.008$), cathepsin D ($P = 0.004$) and SPF ($P = 0.023$) as significant for DFS. All other factors failed to be of significant prognostic value. In a Cox model, only PAI-1 was significant for DFS ($P < 0.001$, relative risk (RR) 6.2). In CART analysis for DFS, the combination of PAI-1 and uPA gave the best risk group discrimination. For OS, PAI-1, cathepsin D, tumour size and ploidy were statistically significant in univariate, but PAI-1 was the only independently significant factor in Cox analysis ($P < 0.001$, RR 8.9). In particular, this analysis shows that PAI-1 is still a strong and independent prognostic factor in node-negative breast cancer after extended 6-year median follow-up.

**Keywords:** breast cancer; plasminogen activator inhibitor type 1 (PAI-1); prognosis; S-phase fraction; tumour biology; urokinase-type plasminogen activator (uPA)

Node-negative breast cancer patients, in contrast to breast cancer patients whose lymph nodes show tumour cell involvement at time of primary therapy, have a low risk of suffering disease recurrences. About 70% of the node-negative patients are cured by surgery alone and will therefore not need any adjuvant systemic therapy. Nevertheless, even within this low-risk breast cancer group, up to 30% of the patients may relapse within 10 years after surgery and eventually die of metastasis (Clark and McGuire, 1988). Traditional histomorphological and clinical factors such as tumour size, tumour grade, steroid hormone receptor status, age, or menopausal status, have been used to identify the high-risk node-negative patients who may benefit from adjuvant systemic therapy (McGuire and Clark, 1992). However, by applying these traditional prognostic factors, more than 75% of all node-negative breast cancer patients will receive adjuvant systemic therapy (McGuire and Clark, 1992), even though only about 30% of all node-negative patients will eventually develop systemic disease. This obvious discrepancy has stimulated the search for new prognostic factors, and numerous factors have been suggested so far for the assessment of breast cancer prognosis (Harris et al, 1992).

Tumour-biological factors such as those reflecting invasion and metastasis or proliferation have strongly been put forward in the literature as new prognostic markers. Several independent investigations have demonstrated that the invasion markers uPA (serine protease urokinase-type plasminogen activator) and PAI-1 (inhibitor of uPA) are statistically independent prognostic factors to predict disease recurrence and death in node-negative breast cancer. Elevated antigen levels of uPA and PAI-1 are associated with poor prognosis (Duffy et al, 1990; Grøhndahl-Hansen et al, 1993; Jänicke et al, 1993; Foekens et al, 1994; Fernö et al, 1996). Another protease, cathepsin D, has also been associated with poor patient outcome (Rochefort, 1992).

Prior to the study of tumour-associated proteolytic factors, flow cytometric DNA analysis was reported to yield valuable prognostic information in breast cancer patients. Ploidy and, in particular, S-phase fraction have been addressed as rather powerful prognostic factors in node-negative breast cancer (Osborne, 1989). In recent years, immunohistochemical detection of the proliferation-associated antigen Ki-67 has also been used for proliferation assessment. The ability to detect Ki-67 in formalin-fixed paraffin sections by means of a monoclonal antibody, MIB1, made this technique readily available for determination of tumour cell proliferation (Dettmar et al, 1997).

Molecular markers, such as the HER-2/neu gene, which codes for an analogue of the epidermal growth factor receptor, as well as the tumour suppressor gene p53, have also been associated with patient prognosis. Yet their prognostic impact is still quite controversial. In addition, their unique tumour-biological role has not yet been fully determined (Clark, 1996).

Even though there is abundant literature on so-called new prognostic factors in primary breast cancer, most publications merely compare one or two new factors to the traditional prognostic

*Received 28 May 1998*
*Revised 21 October 1998*
*Accepted 19 November 1998*

*Correspondence to:* N Harbeck factors. In addition, subgroup analyses of clinically relevant patient collectives such as node-negative patients are often not performed due to small patient numbers. Therefore, after a long-term median follow-up of more than 6 years, we have now evaluated the prognostic impact of eight tumour-biological factors (uPA, PAI-1, cathepsin D, S-phase, ploidy, Ki-67, p53, HER-2/neu) in a homogeneous, clinically important cohort of node-negative patients whose follow-up data were not altered by effects of any adjuvant systemic therapy. In order to ensure comparability of the results, we performed analysis of five factors (S-phase, ploidy, Ki-67, HER-2/neu, p53) on adjacent paraffin sections of the same tissue block. The prognostic impact of the tumour-biological factors on disease-free, as well as overall, survival was compared to that of the traditional prognostic factors tumour size, grading and steroid hormone receptor status.

### MATERIALS AND METHODS

### Patients

Traditional factors (pathological tumour size, steroid hormone receptor status, grading) and tumour-biological factors (uPA, PAI-1, cathepsin D, S-phase, ploidy, Ki-67, p53, HER-2/neu) were assessed in tumour tissues obtained from 125 patients with node-negative breast cancer. Histological grade was scored according to Elston and Ellis (1991); completely undifferentiated tumours in which a histological subtype could not be determined were classified as G4.

Patients either had a modified radical mastectomy ($n = 83$) or underwent breast-conserving surgery with subsequent breast irradiation ($n = 42$) at the Department of Obstetrics and Gynecology of the Technische Universität München, Germany, between 1987 and 1991. In accordance with the standard treatment at the time, none of the patients received any adjuvant systemic therapy. Median age of all patients at primary therapy was 56 years (range 35–82 years). Further patient characteristics are displayed in Table 1. At time of primary therapy no patient had clinical or X-ray evidence of distant metastases. Follow-up data was obtained every 3–6 months. Median follow-up of patients still alive at time of analysis was 76 months (range 47–108 months). Twenty-three patients (18.4%) relapsed. Fifteen patients (12%) died of breast cancer and eight patients died of causes not related to breast cancer within the follow-up period.

**Table 1** Node-negative breast cancer patients without adjuvant systemic therapy. Prospective analyses were performed in all 125 patients, retrospective analyses only in cases where sufficient tumour tissue was left for analysis

| Factors | *n* | (%) |
|---|---|---|
| Tumour size (cm) | 125 | |
| ≤2 | 65 | (52) |
| > 2 and ≤ 5 | 56 | (44.8) |
| > 5 | 4 | (3.2) |
| Steroid hormone receptor status | 125 | |
| Positive | 99 | (79.2) |
| Negative | 26 | (20.8) |
| Grading | 125 | |
| G 1/2 | 93 | (74.4) |
| G 3/4 | 32 | (25.6) |
| PAI-1 | 125 | |
| Low (≤ 14 ng mg$^{-1}$ protein) | 99 | (79.2) |
| High (> 14 ng mg$^{-1}$ protein) | 26 | (20.8) |
| uPA | 125 | |
| Low (≤ 3 ng mg$^{-1}$ protein) | 83 | (66.4) |
| High (> 3 ng mg$^{-1}$ protein) | 42 | (33.6) |
| Cathepsin D | 121 | |
| Low (≤ 41 pmol mg$^{-1}$ protein) | 60 | (49.6) |
| High (> 41 pmol mg$^{-1}$ protein) | 61 | (50.4) |
| S-phase fraction | 101 | |
| Low (≤ 6%) | 55 | (54.5) |
| High (> 6%) | 46 | (45.5) |
| Ploidy | 101 | |
| Diploid (near diploid, diploid) | 51 | (50.5) |
| Aneuploid (an-, multi-, tetraploid) | 50 | (49.5) |
| MIB1 | 116 | |
| Low (≤ 25%) | 96 | (82.8) |
| High (> 25%) | 20 | (17.2) |
| p53 | 111 | |
| Negative | 102 | (91.9) |
| Positive | 9 | (8.1) |
| HER-2/neu | 101 | |
| Negative | 55 | (54.5) |
| Positive | 46 | (45.5) |

### Methods

As described earlier (Jänicke et al, 1990, 1994*a*), uPA and PAI-1 have been measured in tumour tissue extracts in a prospective fashion since 1987 for all breast cancer patients who had their primary surgery performed at our department. uPA and PAI-1 antigen were determined by commercially available enzyme-linked immunosorbent assays (ELISAs) in detergent extracts of breast cancer tissue specimens and expressed as ng of antigen per mg of tissue protein (uPA: Imubind # 894; PAI-1: Imubind # 821; both from American Diagnostica, Greenwich, CT, USA) (Jänicke et al, 1993, 1994*a*; Schmitt et al, 1997*b*). Levels of the protease cathepsin D were determined in the cytosol fraction by ELSA (CIS Bioindustries, Gif-sur-Yvette, France). Total S-phase fraction (SPF) and ploidy were measured by flow cytometry in routinely formalin-fixed, paraffin-embedded tissue sections processed according to Harbeck et al (1994) and then calculated using the computer program ModFit (Verity Software House, ME, USA) (Dettmar et al, 1997).

Immunostaining for p53, HER-2/neu (c-erbB-2) and Ki-67 (MIB1 antibody) was performed as described (Dettmar et al, 1997; Harbeck et al, 1998) in adjacent 4-μm-thick paraffin sections using the alkaline phosphatase anti-alkaline phosphatase (APAAP) method.

### Statistical analysis

Correlations between continuous variables were analysed using the Spearman rank test. Associations between continuous and/or categorical variables were analysed using the Mann–Whitney *U*-test or the $\chi^2$ test as appropriate.

Determination of optimal cut-offs for dichotomized variables to discriminate low-risk and high-risk patients was performed using log-rank statistics. Univariate analyses for disease-free survival were performed according to Kaplan–Meier (Kaplan and Meier, 1958; Jänicke et al, 1993) and univariate Cox analysis. Corrections

 © Cancer Research Campaign 1999

**Table 2** Summary measures for tumour-biological factors

| Factor (units) | uPA (ng mg$^{-1}$ protein) | PAI-1 (ng mg$^{-1}$ protein) | S-phase (%) | Ki-67 (%) | p53 (%) | HER-2/neu (%) | Cathepsin D (pmol mg$^{-1}$ protein) |
|---|---|---|---|---|---|---|---|
| Minimum | 0.07 | 0.06 | 1.08 | 0 | 0 | 0 | 10.6 |
| 1st quartile | 0.86 | 4.11 | 3.27 | 1 | 0 | 0 | 25.67 |
| Median | 1.79 | 7.53 | 5.68 | 10 | 0 | 0 | 41.06 |
| 3rd quartile | 3.67 | 13.19 | 9.53 | 20 | 0 | 10 | 66.67 |
| Maximum | 15.17 | 77.07 | 32.75 | 90 | 70 | 90 | 186.13 |

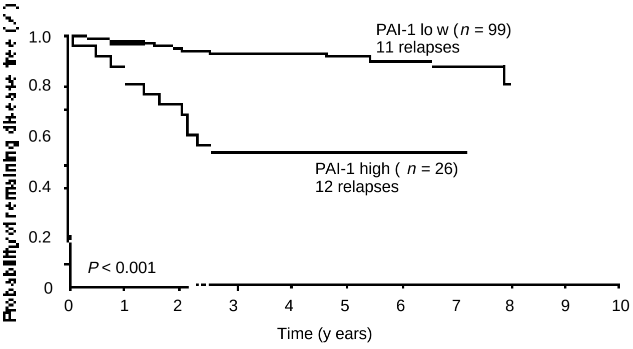

**Figure 1** PAI-1

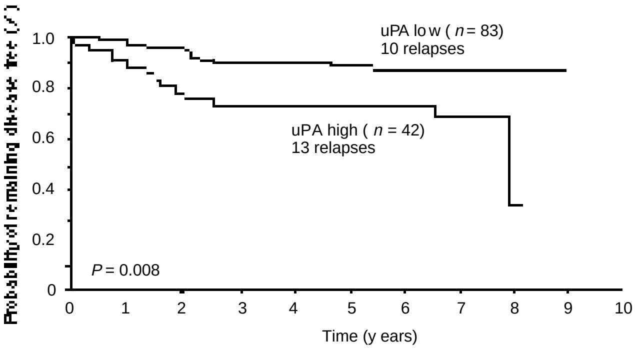

**Figure 3** uPA

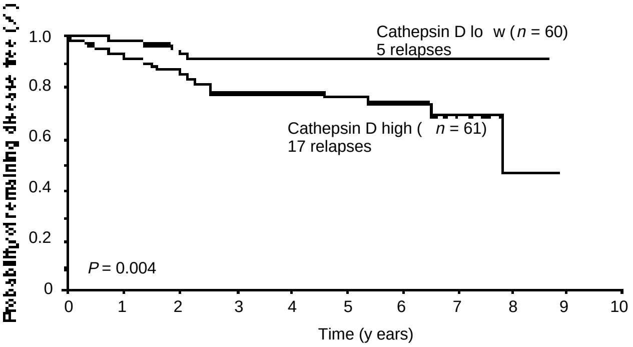

**Figure 2** Cathepsin D

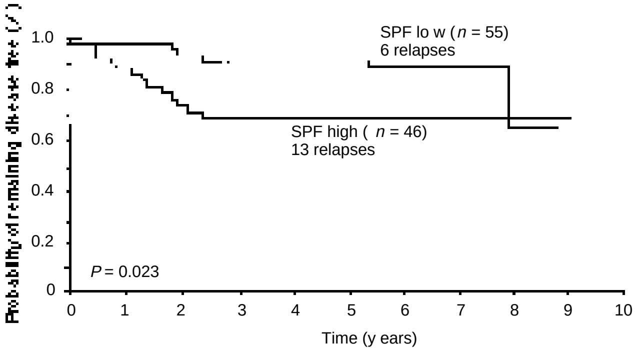

**Figure 4** S-phase fraction (SPF)

**Figures 1–4** Tumour-biological factors and their impact on disease-free survival (DFS) in node-negative breast cancer patients without adjuvant systemic therapy to the *P*-values obtained in Kaplan–Meier analysis to account for multiple testing were computed according to the procedure of Hilsenbeck and Clark (1996).

Multivariate analyses were performed in a forward stepwise fashion by applying the Cox proportional hazards model (Cox, 1972) using the SPSS software package (SPSS Inc., Chicago, IL, USA) and by the CART (classification and regression trees) technique (Breiman et al, 1984; Schmitt et al, 1997*b*). All established and new tumour-biological factors were included in the multivariate analysis. All tests were performed at a significance level of $\alpha = 0.05$ (i.e. with a confidence interval (CI) of 95%).

### RESULTS

### Factors and optimized cut-off values

In 125 node-negative breast cancer patients who did not receive any adjuvant systemic therapy, traditional histomorphological prognostic factors, as well as invasion markers uPA and PAI-1, were prospectively determined. Additional tumour-biological factors (cathepsin D, S-phase fraction, ploidy, Ki-67, HER-2/neu, p53) were retrospectively determined in all cases with a sufficient amount of tumour tissue left for analysis (Table 1). Multivariate

© Cancer Research Campaign 1999

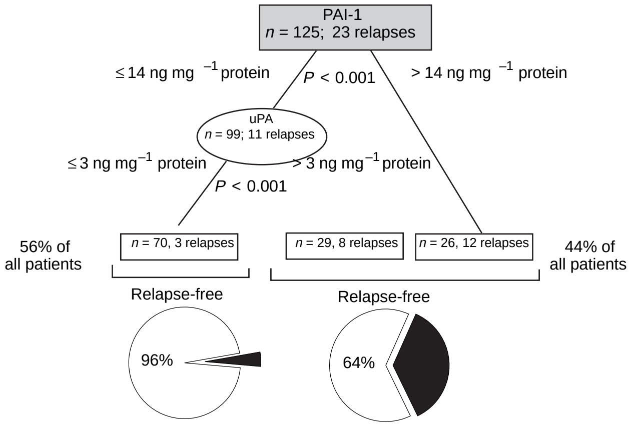

Figure 5 CART analysis for disease-free survival in node-negative breast cancer patients without adjuvant systemic therapy, performed at a median follow-up of 76 months

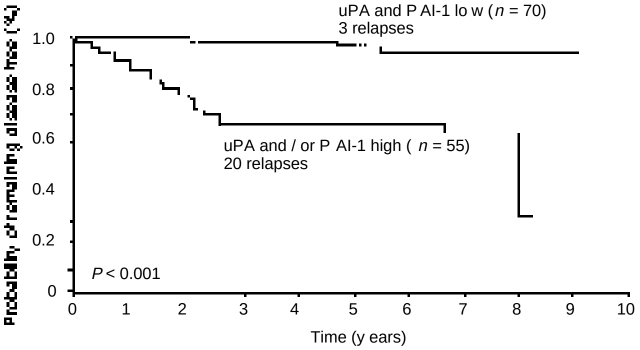

Figure 6 The combination of uPA and PAI-1 and its impact on disease-free survival (DFS) in node-negative breast cancer patients analyses were performed including those 96 patients for whom all factors were available. Tumour-biological as well as traditional factors were then related to patient outcome with a median follow-up period of 76 months. To give an indication of the statistical distributions of the tumour-biological factors, we report the median and quartiles in Table 2.

The following cut-off values were optimized using log-rank statistics and assigned for uPA (3 ng $mg^{-1}$ protein), PAI-1 (14 ng $mg^{-1}$ protein), total S-phase fraction (6%), Ki-67 (25%), p53 (negative vs positive), HER-2/neu (2.5%; i.e. negative vs positive), and cathepsin D (41 pmol $mg^{-1}$ protein) (Harbeck et al, 1998). Ploidy was coded as a binary variable into diploid (diploid and near diploid) and aneuploid (an-, multi-, tetraploid) (Dettmar et al, 1997).

### Associations and correlations

The following associations between tumour-biological and traditional factors were significant ($P < 0.05$): uPA: grading only; PAI-1: grading, hormone receptors, tumour size; p53: grading, hormone receptors; Ki-67: grading only; S-phase: hormone receptors, tumour size; ploidy: age only. Correlation co-efficients between tumour-biological factors were assessed, and no strong correlation ($r > 0.50$) between any of these factors was observed. The following correlation co-efficients were significant: PAI-1 and uPA ($r = 0.325$); cathepsin D and uPA ($r = 0.272$); cathepsin D and PAI-1 ($r = 0.228$); Ki-67 (MIB1) and p53 ($r = 0.314$); S-phase and uPA ($r = 0.321$); S-phase and PAI-1 ($r = 0.218$). As for the two proliferation markers analysed, a significant correlation was only seen between Ki-67 (MIB1) and aneuploid SPF ($P = 0.011$, $r = 0.383$). Ploidy was significantly associated with S-phase.

### Univariate and multivariate analyses

In univariate analysis, PAI-1 ($P < 0.001$), cathepsin D ($P = 0.004$), uPA ($P = 0.008$) and SPF ($P = 0.023$) are significantly associated with disease-free survival (DFS) (Figures 1–4). Adjusting the $P$-values according to Hilsenbeck and Clark (1996) yields PAI-1 ($P < 0.001$), cathepsin D ($P = 0.08$), uPA ($P = 0.16$) and SPF ($P = 0.32$); quantitative analysis without dichotomization yields PAI-1 ($P < 0.001$), cathepsin D ($P = 0.065$), uPA ($P = 0.007$) and SPF ($P = 0.055$). Tumour size, steroid hormone receptor status, grading, p53, HER-2/neu and Ki-67 (MIB1) have no significant prognostic impact on DFS in our group of patients after a median follow-up of more than 6 years. In multivariate analysis, only PAI-1 ($P < 0.001$, relative risk (RR) 6.2; 95% CI 2.3–16.4) is of independent statistical significance for DFS.

A CART analysis for DFS was performed including all traditional and tumour-biological factors (Figure 5). It shows PAI-1 to be the strongest factor for risk group selection ($P < 0.001$). Patients with high PAI-1 levels (> 14 ng $mg^{-1}$ protein) in their primary tumour belong to a high-risk group ($n = 26$, 12 relapses) for which no other prognostic factor was able to achieve a significantly better sub-classification. In contrast, among patients with low PAI-1 levels (≤ 14 ng $mg^{-1}$ protein), uPA turned out to be an additional strong selection factor ($P < 0.001$), allowing patients to be subdivided into groups with low PAI-1 and low or high uPA antigen levels (≤/> 3 ng $mg^{-1}$ protein) in their primary tumours. As a result, 56% of all patients belong to a low-risk group ($n = 70$) defined by low PAI-1 and low uPA levels encompassing only three relapses (4%). Among the remaining 55 patients (44% of the total 125 patients) having high levels of PAI-1 or uPA, 20 relapses (36%) are found within the follow-up period (Figures 5 and 6).

In order to compare our data within the time frame used in clinical practice, we also looked at the 5-year relapse rates (Figure 7). Patients with high PAI-1 levels in their tumours have the highest 5-year relapse rate (47%). However, the combination of uPA and PAI-1 gives the best definition of a low-risk group with a very low 3% relapse rate at 5 years. PAI-1, SPF and cathepsin D all have a 5-year relapse rate of 8% within their respective low-risk groups. All other factors have an 11% or even higher 5-year relapse rate within their low-risk set of patients. There was no qualitative difference between 5-year relapse rates as estimated by Kaplan–Meier analysis and relapse rates at the end of the follow-up period (median 76 months). In conclusion, 20 of all 23 relapses (87%) within the follow-up period were correctly classified by elevated PAI-1 and/or uPA antigen levels determined in the tumour tissue extracts at time of primary therapy.

For overall survival (OS), a similar picture emerged. PAI-1 proved to be a statistically significant independent prognostic factor in both univariate and multivariate analysis ($P < 0.001$, RR 8.9; 95% CI 3.7–21.9). In addition, cathepsin D ($P = 0.010$), tumour size ($P = 0.035$) and ploidy ($P = 0.049$) were significant

 © Cancer Research Campaign 1999

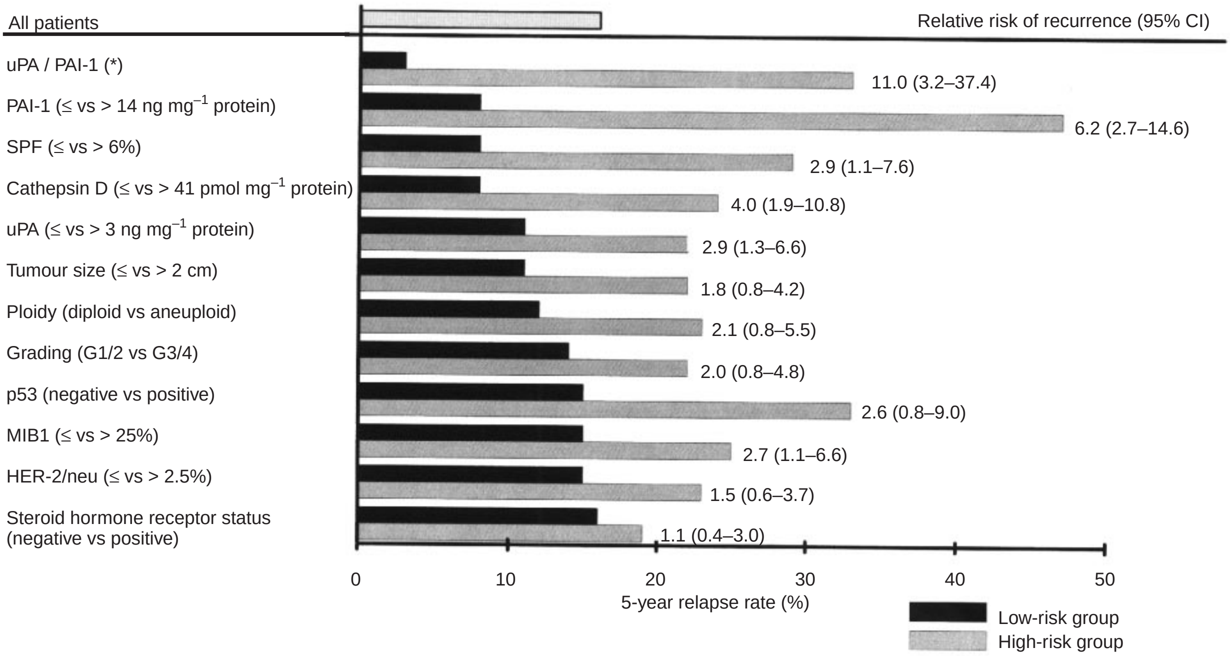

**Figure 7** Five-year relapse rate in node-negative breast cancer patients without adjuvant systemic therapy was estimated by Kaplan–Meier analysis. Relative risks of relapse and the 95% confidence intervals between the respective risk groups (high-risk vs low-risk group as determined by the respective prognostic factor mentioned on the left) were calculated by univariate Cox analysis. *uPA/PAI-1: both factors low vs either or both factors high in univariate but not in multivariate analysis. The adjusted *P*-values (Hilsenbeck and Clark, 1996) for PAI-1 were $P < 0.001$ and for cathepsin $P = 0.18$. All other tumour-biological and traditional factors had no significant prognostic impact on OS.

### DISCUSSION

Of the eight tumour-biological factors investigated, only the three factors describing the invasive and metastatic capacity of the tumour (uPA, PAI-1, cathepsin D), and S-phase fraction (SPF), a marker reflecting its proliferative potential, yielded statistically significant prognostic information in node-negative breast cancer patients. In contrast, the traditional prognostic factors (tumour size, steroid hormone receptor status and grading) were of no value to predict DFS or OS. After weighting the few significant factors by multivariate analysis, only PAI-1 turned out to be of statistically independent prognostic significance. This strong prognostic impact of PAI-1 is also reflected by the low univariate *P*-values even after adjustment for multiple testing as well as in quantitative analysis.

In two earlier papers, after median follow-up periods of less than 3 years, our group was the first to show that both PAI-1 and uPA were significant prognostic factors in multivariate analysis for DFS in both node-positive and node-negative breast cancer patients (Jänicke et al, 1991, 1993). These results were subsequently confirmed by others (Grøhndahl-Hansen et al, 1993; Bouchet et al, 1994; Foekens et al, 1994; Grøhndahl-Hansen et al, 1997*a*, 1997*b*) after median follow-up periods ranging from 4 (Foekens et al, 1994) to more than 8 years (Grøhndahl-Hansen et al, 1993). All of these groups used cytosol preparations instead of detergent extracts for determination of uPA and PAI-1 antigen levels. Cytosols give comparable results for PAI-1 but may not detect the full prognostic impact of uPA due to lower antigen determination levels (Jänicke et al, 1994*a*). Statistical analyses performed in our group of patients at different times of follow-up suggested a variation of the prognostic strength of uPA and PAI-1 with time. The prognostic impact of uPA seems to be most pronounced during the first 2 years after primary therapy, whereas that of PAI-1 actually increased (Schmitt et al, 1997*b*). As shown in the present publication, PAI-1 prevails as a strong prognostic factor in node-negative breast cancer patients at a median follow-up of more than 6 years. Moreover, this long-term follow-up confirms our earlier findings that the combination of uPA and PAI-1 is well suited to select a group of patients having a very low risk of relapse (Jänicke et al, 1993, 1994*b*). In the low-risk group (both uPA and PAI-1 low) after a median follow-up of more than 6 years, 96% of the patients remain relapse-free, in contrast to only 64% of patients in the high-risk group (either or both factors high). Patients with either or both factors high have an 11-times higher risk of recurrence than patients with both factors low. To our knowledge, such a clear-cut risk-group separation has not been demonstrated for any of the traditional factors, and indeed not for any other tumour-biological factor. Independent validation using additional patient data will be a very important next step.

Foekens et al (1994) presented similar data for a group of primary breast cancer patients, including node-negative and node-positive patients, and showed that patients with low levels of both uPA and PAI-1 had a better prognosis than patients with either or both factors high. Unfortunately, other groups who studied uPA and PAI-1 in breast cancer have not looked at the prognostic impact of the combination of the two factors. Definition of a low-risk group having a very low risk of disease recurrence is,

© Cancer Research Campaign 1999 

however, a prerequisite for any therapy recommendation considering prognostic factors. Based on our earlier results concerning uPA, PAI-1 and breast cancer prognosis, a prospective multicentre therapy trial was started in Germany in mid 1993 in which node-negative patients with high uPA and/or PAI-1 antigen levels in their primary tumours are randomized for adjuvant standard CMF chemotherapy or observation only (Jänicke et al, 1994*b*). This trial addresses two main issues: (1) Can the strong prognostic impact of uPA and PAI-1 be confirmed in a prospective multicentre study and (2) do uPA and/or PAI-1 have a predictive value with regard to administering CMF chemotherapy? At present, about 650 patients are enrolled in this trial. We want to stress that up to now no contradictory data regarding the prognostic impact of uPA and PAI-1 in primary breast cancer have been reported in the literature.

Over the last few years, data from basic research have accumulated that may help to explain the clinical finding that not only high levels of the protease uPA, but also high levels of its inhibitor PAI-1, in the primary tumour tissue indicate poor patient outcome. Whereas uPA facilitates metastasis by degradation of extracellular matrix (Danø et al, 1985; Schmitt et al, 1997*a*), PAI-1 may play another important role in tumour biology apart from being an inhibitor of uPA. After interaction of PAI-1 with uPA already complexed with the uPA receptor (uPAR), this ternary complex is internalized into the cell, thereby initiating signal transduction and cell proliferation. Moreover, PAI-1 acts as an inhibitor of cell adhesion by interfering with attachment of the tumour cell to the extracellular matrix component vitronectin (Stefansson et al, 1996; Wei et al, 1996). Interestingly, binding of uPA to PAI-1 may reverse this process and support cell adhesion and migration (Lauffenburger, 1996).

In addition to uPA and PAI-1, the protease cathepsin D also has a significant prognostic impact on DFS and OS in our group of patients as assessed by univariate analysis. This is quite consistent with our earlier data (Jänicke et al, 1993). Foekens et al (1994) also reported a prognostic impact of cathepsin D on DFS which did not persist, however, in the presence of uPA and PAI-1 in multivariate analysis. Spyratos et al (1992) also found uPA to be significant in both univariate and multivariate analysis, whereas cathepsin D just failed significance. Unfortunately, as shown in many investigations, methodological differences and heterogeneous patient collectives have rendered the data regarding the prognostic impact of cathepsin D quite controversial (Ravdin, 1993).

SPF, a proliferation marker, also showed a significant prognostic impact in our set of node-negative patients after a median follow-up of 76 months. This confirms earlier observations, where we found after a median follow-up of 37.5 months that, even though both SPF and Ki-67 were significant prognostic factors for DFS in node-negative breast cancer patients, only SPF remained significant in multivariate analysis (Dettmar et al, 1997). In the present study, in a homogeneous group of node-negative patients who did not receive any adjuvant systemic therapy at a considerably longer median follow-up period of more than 6 years, Ki-67 was not able to keep its prognostic significance. Other researchers who compared SPF and Ki-67 also found SPF to give better prognostic information, because Ki-67 either lost its univariate significance in multivariate analysis (Gasparini et al, 1994) or was the weaker factor in multivariate analysis (Brown et al, 1996). Wenger et al (1993) published on a large data set and showed that SPF in breast cancer has a significant prognostic impact on DFS in node-negative patients ($n = 9736$). There is a general agreement in the literature that proliferation markers, and in particular SPF, provide valuable information in breast cancer prognosis (Clark, 1996). Their clinical usefulness could be greatly enhanced if methodological standardization were performed. There are very few reports comparing proliferation markers with other tumour-biological markers, in particular with invasion markers. In our study, a statistically independent prognostic impact of the proliferation marker SPF could not be demonstrated when the invasion and metastasis markers uPA and PAI-1 were included in the analysis. This may be partly due to the fact that there are significant correlations between uPA/PAI-1 and SPF – a clinical finding that corresponds well with experimental evidence suggesting that cell proliferation is stimulated after internalization of the uPA/PAI-1/uPA-R complex.

Ploidy was not a statistically significant factor for DFS and only of borderline significance for OS. Although there is considerable (yet controversial) data concerning the importance of ploidy in breast cancer prognosis (McGuire and Clark, 1992), most authors seem to agree on the fact that, if at all, ploidy is of low prognostic power.

HER-2/neu and p53 did not contribute any significant prognostic information in our set of node-negative patients. We note that the literature regarding these two factors is still very much in disagreement concerning their prognostic value (Clark, 1996). For HER-2/neu, both detection of protein overexpression by immunohistochemistry as well as immunoblotting for detection of gene amplification have been used in the past to study its prognostic impact. A new approach, detection of HER2/neu gene amplification by fluorescence in situ hybridization (FISH), has recently been introduced to identify high-risk node-negative breast cancer patients (Press et al, 1997) and seems to be superior to the immunohistochemical approach. As for p53, it is still under discussion whether mutant p53 and/or wild-type p53 are associated with the malignant phenotype.

In conclusion, substantial evidence has accumulated that the invasion and metastasis markers uPA and PAI-1, as well as the proliferation marker SPF, are able to generate clinically relevant prognostic information in node-negative breast cancer patients. Serious efforts towards international standardization, particularly for uPA and PAI-1 determination, have been recently undertaken (Benraad et al, 1996). Similar quality control studies have been introduced for flow cytometric DNA analysis (D'hautcourt et al, 1996), and international guidelines for DNA flow cytometry were proposed by the International Society for Analytical Cytology (ISAC) (Hiddemann et al, 1984). Reliable and standardizable SPF calculation has been made possible by modern evaluation software in both paraffin and fresh tumour tissue (Bagwell et al, 1991). Consequently, uPA, PAI-1 and SPF seem to be the tumour-biological prognostic markers that are most suited for transfer into clinical practice (Graeff et al, 1997). Recommendations of the EORTC Receptor and Biomarker Study Group to include these factors into the routine panel for breast cancer patient assessment were put forward in 1995 (Blankenstein, 1997). Additional studies comparing several of these tumour-biological prognostic factors in homogeneous patient cohorts with sufficient follow-up periods are needed. Together with preliminary results of the German prospective therapy trial, they will help not only to further determine the prognostic impact of uPA and PAI-1 (and facultative tumour-biological factors) in node-negative breast cancer, but also to evaluate their predictive value with regard to therapy response.

© Cancer Research Campaign 1999

### ACKNOWLEDGEMENTS

This work was supported by the Deutsche Forschungsgemeinschaft (Clinical Research Group GR 280/4-1/2/5, German Research Foundation) and the BIOMED-1 project BMH1-CT93-1346 of the European Union.

### REFERENCES

Bagwell CB, Mayo SW, Whetstone SD, Hitchcox SA, Baker DR, Herbert DJ, Weaver DL, Jones MA and Lovett EJ (1991) DNA histogram debris theory and compensation. *Cytometry* **12**: 107–118

Benraad TJ, Geurts-Moespot J, Grøndahl-Hansen J, Schmitt M, Heuvel JJTM, deWitte JH, Foekens JA, Leake RE, Brünner N and Sweep CGJ (1996) Immunoassays (ELISA) of urokinase-type plasminogen activator (uPA): Report of an EORTC/BIOMED-1 workshop. *Eur J Cancer* **32A**: 1371–1381

Blankenstein MA (1997) Biochemical assessment of tissue prognostic factors in breast cancer. *Breast* **6**: 31–37

Bouchet C, Spyratos F, Martin PM, Hacène K, Gentile A and Oglobine J (1994) Prognostic value of urokinase-type plasminogen activator (uPA) and plasminogen activator inhibitors PAI-1 and PAI-2 in breast carcinomas. *Br J Cancer* **69**: 398–405

Breiman I, Friedman J, Olshen R and Stone C (1984) *Classification and Regression Trees*. Chapman and Hall: New York

Brown RW, Allred CD, Clark GM, Osborne C and Hilsenbeck S (1996) Prognostic value of Ki-67 compared to S-phase fraction in axillary node-negative breast cancer. *Clin Cancer Res* **2**: 585–592

Clark GM (1996) Prognostic and predictive factors. In: *Diseases of the Breast*. Harris JR, Lippmann ME, Morrow M and Hellmann S (eds), pp. 461–485. Lippincott-Raven: Philadelphia

Clark GM and McGuire W (1988) Steroid receptors and other prognostic factors in primary breast cancer. *Semin Oncol* **15**: 20–25

Cox DR (1972) Regression models and life-tables. *J R Stat Soc (B)* **34**: 187–200

Danø K, Andreasen PA, Grøhndahl-Hansen J, Kristensen P, Nielsen L and Skriver L (1985) Plasminogen activators, tissue degradation and cancer. *Adv Cancer Res* **44**: 139–266

Dettmar P, Harbeck N, Thomssen C, Pache L, Ziffer P, Fizi K, Jänicke F, Nathrath W, Schmitt M, Graeff H and Höfler H (1997) Prognostic impact of the proliferation associated factors Ki-67 and S-phase in node-negative breast cancer using MIB1 immunostaining and flowcytofluorometric S-phase determination. *Br J Cancer* **75**: 1525–1533

D'hautcourt JL, Spyratos F and Chassevent A, on behalf of the Association Francaise de Cytometrie (1996) Quality control study by the French cytometry association on flow cytometric DNA content and S-phase fraction (S%). *Cytometry (Comm Clin Cytometry)* **26**: 32–39

Duffy MJ, Reilley D, O'Sullivan C, O'Higgins N, Fennelly JN and Andreasen P (1990) Urokinase-plasminogen activator, a new and independent prognostic marker in breast cancer. *Cancer Res* **50**: 6827–6829

Elston CW and Ellis IO (1991) Pathological prognostic factors in breast cancer. I. The value of histological grade in breast cancer: experience from a large study with long-term follow-up. *Histopathology* **19**: 403–410

Fernö M, Bendahl PO, Borg A, Brundell J, Hirschberg L, Olsson H and Killander D (1996) Urokinase plasminogen activator, a strong independent prognostic factor in breast cancer, analysed in steroid receptor cytosols with a luminometric immunoassay. *Eur J Cancer* **32A**: 793–801

Foekens JA, Schmitt M, van Putten WLJ, Peters HA, Kramer MD, Jänicke F and Klijn JGM (1994) Plasminogen activator inhibitor-1 and prognosis in primary breast cancer. *J Clin Oncology* **12**: 1648–1658

Gasparini G, Boracchi P, Verderio P and Bevilacqua P (1994) Cell kinetics in human breast cancer: Comparison between the prognostic value of the cytofluorimetric S-phase fraction and that of the antibodies to Ki-67 and PCNA antigens detected by immunocytochemistry. *Int J Cancer* **57**: 822–829

Graeff H, Wilmanns W, Jänicke F, Sauer H and Classen S (1997) Prognostische und therapierelevante Faktoren beim Mammakarzinom. Ergebnisse einer Konsensuskonferenz. *Onkologe* **3**: 409–412

Grøhndahl-Hansen J, Christensen IJ, Rosenquist C, Brünner N, Mouridsen HT, Danø K and Blichert-Toft M (1993) High levels of urokinase-type plasminogen activator and its inhibitor PAI-1 in cytosolic extracts of breast carcinomas are associated with poor prognosis. *Cancer Res* **53**: 2513–2521

Grøhndahl-Hansen J, Christensen IJ, Briand P, Pappot H, Mouridsen HT, Blichert-Toft M, Danø K and Brünner N (1997*a*) Plasminogen activator inhibitor type 1 in cytosolic tumor extracts predicts prognosis in low-risk breast cancer patients. *Clin Cancer Res* **3**: 233–239

Grøhndahl-Hansen J, Hilsenbeck SG, Christensen IJ, Clark GM, Osborne CK and Brünner N (1997*b*) Prognostic significance of PAI-1 and uPA in cytosolic extracts obtained from node-positive breast cancer patients. *Breast Cancer Res Treat* **43**: 153–163

Harbeck N, Yamamoto N, Moniwa N, Schüren E, Ziffer P, Dettmar P, Höfler H, Schmitt M and Graeff H (1994) Flow cytometric DNA analysis in primary breast cancer: technical pitfalls and clinical applications. In: *Prospects in Diagnosis and Treatment of Cancer*, Schmitt M, Graeff H and Jänicke F (eds), pp. 63–74. Elsevier Science: Amsterdam

Harbeck N, Dettmar P, Thomssen C, Henselmann B, Kuhn W, Ulm K, Jänicke F, Höfler H, Graeff H and Schmitt M (1998) Prognostic impact of tumor biological factors on survival in node-negative breast cancer. *Anticancer Res* **18**: 2187–2198

Harris JR, Lippman ME, Veronesi U and Willett W (1992) Medical progress: breast cancer (third of three parts). *N Engl J Med* **327**: 473–490

Hiddemann W, Schumann J, Andreeff M, Barlogie B, Herman CJ, Leif RC, Mayall BH, Murphy RF and Sandberg AA (1984) Convention on nomenclature for DNA cytometry. *Cytometry* **5**: 445–446

Hilsenbeck SG and Clark GM (1996) Practical p-value adjustment for optimally selected cutpoints. *Stat Med* **15**: 103–112

Kaplan EL and Meier P (1958) Non-parametric estimation from incomplete observations. *J Am Stat Assoc* **53**: 457–481

Jänicke F, Schmitt M, Hafter R, Hollrieder A, Babic R, Ulm K, Gössner W and Graeff H (1990) Urokinase-type plasminogen activator (uPA) antigen is a predictor of early relapse in breast cancer. *Fibrinolysis* **4**: 69–78

Jänicke F, Schmitt M and Graeff H (1991) Clinical relevance of the urokinase-type and tissue-type plasminogen activators and of their type 1 inhibitor in breast cancer. *Semin Thromb Hemostasis* **17**: 303–312

Jänicke F, Schmitt M, Pache L, Ulm K, Harbeck N, Höfler H and Graeff H (1993) Urokinase (uPA) and its inhibitor PAI-1 are strong, independent prognostic factors in node-negative breast cancer. *Breast Cancer Res Treat* **24**: 195–208

Jänicke F, Pache L, Schmitt M, Ulm K, Thomssen C, Prechtl A and Graeff H (1994*a*) Both the cytosols and detergent extracts of breast cancer tissues are suited to evaluate the prognostic impact of the urokinase-type plasminogen activator and its inhibitor, plasminogen activator inhibitor type 1. *Cancer Res* **54**: 2527–2530

Jänicke F, Thomssen C, Pache L, Schmitt M and Graeff H (1994*b*) Urokinase (uPA) and PAI-1 as selection criteria for adjuvant chemotherapy in axillary node-negative breast cancer patients. In *Prospects in Diagnosis and Treatment of Cancer*, Schmitt M, Graeff H and Jänicke F (eds), pp. 207–218. Elsevier Science: Amsterdam

Lauffenburger D (1996) Making connections count. *Nature* **383**: 390–391

McGuire W and Clark GM (1992) Prognostic factors and treatment decisions in axillary node-negative breast cancer. *N Engl J Med* **326**: 1756–1761

Osborne CK (1989) DNA flow cytometry in early breast cancer: a step in the right direction. *J Natl Cancer Inst* **81**: 1344–1345

Press MF, Bernstein L, Thomas PA, Meisner LF, Zhou JY, Ma Y, Hung G, Robinson RA, Harris C, El-Naggar A, Slamon DJ, Phillips RN, Ross JS, Wolman SR and Flom KJ (1997) HER-2/neu gene amplification characterized by fluorescence in situ hybridization: poor prognosis in node-negative breast carcinomas. *J Clin Oncol* **15**: 2894–2904

Ravdin PM (1993) Evaluation of cathepsin D as a prognostic factor in breast cancer. *Breast Cancer Res Treat* **24**: 219–226

Rochefort H (1992) Cathepsin D in breast cancer: a tissue marker associated with metastasis. *Eur J Cancer* **28A**: 1780–1783

Schmitt M, Jänicke F and Graeff H (1992) Tumor-associated proteases. *Fibrinolysis* **6**: 3–26

Schmitt M, Harbeck N, Thomssen C, Wilhelm O, Magdolen V, Reuning U, Ulm K, Höfler H, Jänicke F and Graeff H (1997*a*) Clinical impact of the plasminogen activation system in tumor invasion and metastasis: prognostic relevance and target for therapy. *Thrombosis Haemostasis* **78**: 285–296

Schmitt M, Thomssen C, Ulm K, Seiderer A, Harbeck N, Höfler H, Jänicke F and Graeff H (1997*b*) Time-varying prognostic impact of tumor biological factors urokinase (uPA), PAI-1, and steroid hormone receptor status in primary breast cancer. *Br J Cancer Inst* **76**: 306–311

Spyratos F, Martin PM, Hacène K, Romain S, Andrieu C, Ferrero-Poüs M, Deytieux S, LeDoussal V, Tubiana-Hulin M and Brunet M (1992) Multiparametric prognostic evaluation of biological factors in primary breast cancer. *J Natl Cancer Inst* **84**: 1266–1272

© Cancer Research Campaign 1999

Stefansson S and Lawrence DA (1996) The serpin PAI-1 inhibits cell migration by blocking integrin $\alpha_v\beta_3$ binding to vitronectin. *Nature* **383**: 441–443
Wei Y, Lukashev M, Simon DI, Bodary SC, Rosenberg S, Doyle MV and Chapman HA (1996) Regulation of integrin function by the urokinase receptor. *Science* **273**: 1551–1555
Wenger CR, Beardslee S, Owens MA, Pounds G, Oldaker T, Vendely P, Pandian MR, Harrington D, Clark GM and McGuire W (1993) DNA ploidy, S-phase, and steroid receptors in more than 127 000 breast cancer patients. *Breast Cancer Res Treat* **28**: 9–20

*© Cancer Research Campaign 1999*
